# Uncovering new signaling proteins and potential drug targets through the interactome analysis of *Mycobacterium tuberculosis*

**DOI:** 10.1186/1471-2164-10-118

**Published:** 2009-03-19

**Authors:** Tao Cui, Lei Zhang, Xizhou Wang, Zheng-Guo He

**Affiliations:** 1 National Key Laboratory of Agricultural Microbiology, Center for Proteomics Research, College of Life Science and Technology, Huazhong Agricultural University, Wuhan 430070, PR China; 2 Department of Medicine, Lishui University, Lishui 323000, PR China

## Abstract

**Background:**

Analysis of the pathogen interactome is a powerful approach for dissecting potential signal transduction and virulence pathways. It also offers opportunities for exploring new drug targets.

**Results:**

In this study, a protein-protein interaction (PPI) network of *Mycobacterium tuberculosis *H37Rv was constructed using a homogenous protein mapping method, which has shown molecular chaperones, ribosomal proteins and ABC transporters to be highly interconnected proteins. A further analysis of this network unraveled the function of hypothetical proteins as well as a potential signaling pathway. A hypothetical protein, Rv2752c, which was linked to a metal cation-transporting ATPase, was characterized as a metal-beta-lactamase, through domain analysis in combination with an *in vitro *activity experiment. A second hypothetical protein, Rv1354c, and an unknown protein kinase, PknK, interacted with a similar group of inner membrane-associated ABC transporters in the PPI network. The interactions of Rv1354 with these proteins were also confirmed by a further bacterial two-hybrid analysis. According to protein domain structures, the unique *M. tuberculosis *Rv1354c gene was proposed, for the first time, to be responsible for the turnover of cyclic-di-GMP, a second messenger molecule in this bacterium. A further structure-based inhibitors screening for Rv1354c was also performed *in silicon*.

**Conclusion:**

We constructed a comprehensive protein-protein interaction network for *M. tuberculosis *consisting of 738 proteins and 5639 interaction pairs. Our analysis unraveled the function of hypothetical proteins as well as a potential signaling pathway. The group of ABC transporters, PknK, and Rv1354c were proposed to constitute a potential membrane-associated signaling pathway that cooperatively responds to environmental stresses in *M. tuberculosis*. The study therefore provides valuable clues in exploring new signaling proteins, virulence pathways, and drug targets.

## Background

The intracellular pathogen *Mycobacterium tuberculosis*, the causative agent of tuberculosis (TB), is responsible for nearly two million human deaths worldwide every year. In addition, one-third of the world's population is currently infected with the TB bacillus. The situation has worsened in recent years with the emergence of multi-drug resistant TB and co-infection with HIV [1]. Understanding the signal transduction behind virulence and the infection mechanisms of *M. tuberculosis *is therefore critical for the identification of new drug targets and the development of new drugs.

The *M. tuberculosis *bacterium confronts a highly hostile environment during infection, including restricted access to nutrients and reduced oxygen tension [2]. Its ability to infect under these conditions has been suggested that it may use unique pathogenic mechanisms to facilitate integrated responses to the multiple stresses it encounters within the phagosome. However, little is known regarding the specific bacterial components involved in this process. Moreover, the molecular mechanism involved in sensing of extracellular signals for inducing its metabolic adaptation still remains unclear.

Protein-protein interactions (PPI) play significant roles in many biological processes, such as in the assembly of molecular complexes or in signal transduction. At a more applied level, protein interaction networks also provide tools for exploration of novel drug targets [3]. However, characterization of large-scale protein interaction networks from most organisms is still problematic due to the expense and lengthy time requirements of these types of studies. However, interaction data based on a high throughput experimental technique from model organisms can be utilized in cases where orthologs of interacting proteins can be clearly identified [4]. Walhout et al. [5] introduced the notion of the 'interolog', orthologous pairs of interacting proteins in different organisms. This idea was further extended to include paralogous interactions [6]. Orthologous interactions, or protein pairs with interacting orthologs, have been used to construct entire interactomes for organisms for which actual experimental interactions are sparse [7-9].

In this study, we constructed an *M. tuberculosis *PPI network consisting of 738 proteins and 5639 interaction pairs using a homogenous protein mapping method. Analysis of this network has provided a number of valuable clues for exploration of new virulence pathways and drug targets.

## Results

### A comprehensive PPI network for *M. tuberculosis*

**Figure 1:**
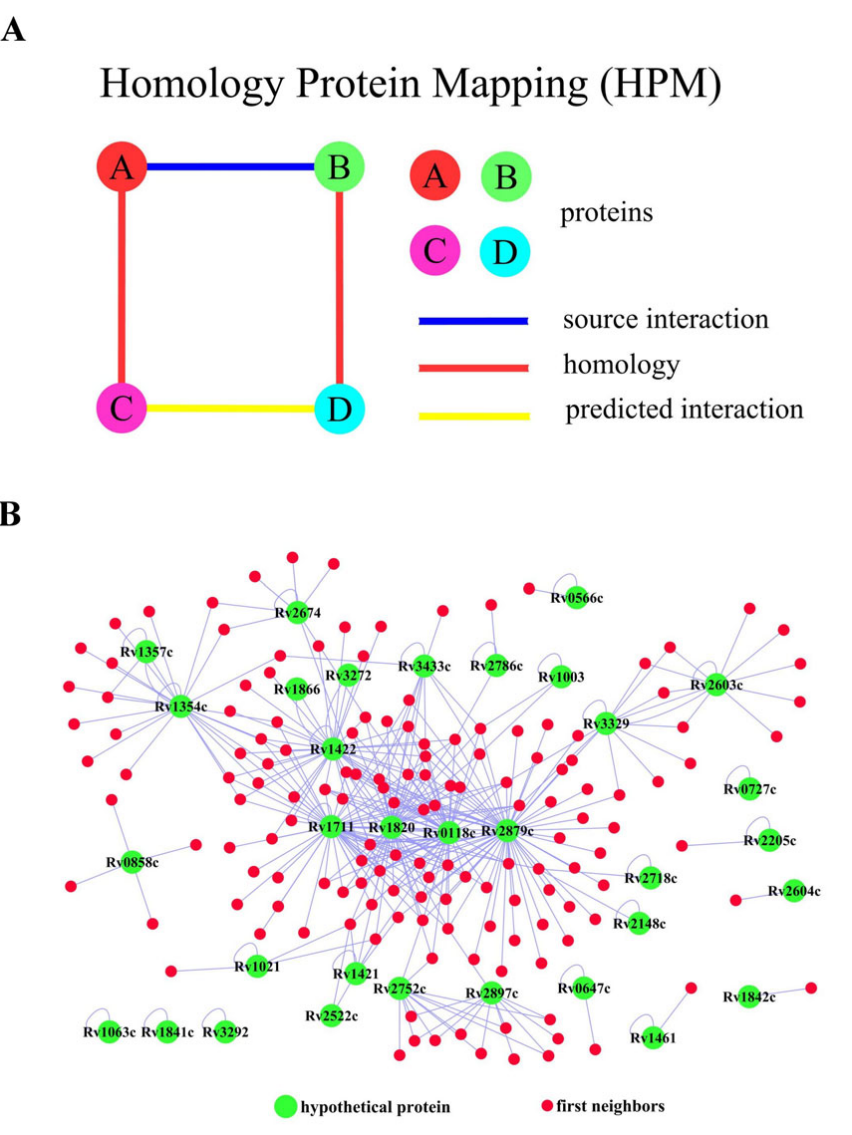
**(A) Schematic diagram of the homologous protein mapping (HPM) method**. Two proteins are predicted to have functional linkage (linked with the yellow line) if they have higher homology (linked with the red line) with a pair of interactive proteins that have been validated experimentally (linked with the blue line). Circles represent different proteins. (**B**) A local PPI network of hypothetical proteins. Thirty-three proteins (green dots) that were annotated as hypothetical proteins in genome annotation appeared in the predictive protein interaction network generated in this study (see Additional file 3).

Based on protein interaction information obtained from high throughput experiments, we have used a computational method called homologous protein mapping (HPM) to predict *M. tuberculosis *protein interactions at the proteomic scale. The rationale is that, for any pair of interactive proteins validated experimentally in the Database of Interaction Protein (DIP) [10], two proteins will be predicted to have a functional linkage if they demonstrate a higher homology. By this method, interaction evidence from model organisms was used when orthologs of interacting proteins could be clearly identified in *M. tuberculosis*. The schematic diagram shown in Fig. 1A is a simplified model for a protein that corresponds to just one other single protein. Using the HPM method, a virtual protein interaction network of *M. tuberculosis *H37Rv was constructed, consisting of 738 proteins and 5639 non-redundant interaction pairs (see Additional file 1).

Several computational methods have recently been developed to predict protein interactions [3,11,12]. Bowers and his co-workers combined these computational algorithms to predict the protein interaction information for a multitude of microorganisms and released all of these data on the ProLinks database [13]. To evaluate the reliability of the PPI network, HPM data were compared with other computational methods (Table 1). The HPM data had a relatively high overlap with data produced by the Rosetta Stone method (RS) [11], although it had a very low overlap with that from the Gene Cluster method (GC) [14]. This phenomenon was also observed between other methods (Table 1), thereby demonstrating that there might be complementarities between the methods.

**Table 1:** Overlaps between various computational predictive methods.

	**GC**(2625)	**GN**(4351)	**PP**(3894)	**RS**(7533)	**HPM**(6091)
**GC**(2625)		483	83	148	**57**
**GN**(4351)	483		567	323	**336**
**PP**(3894)	83	567		690	**562**
**RS**(7533)	148	323	690		**985**

GC, Operon/Gene Cluster method; GN, Conserved Gene methods; PP, Phylogenetic Profile method; RS, Rosetta Stone method; HPM, homogenous protein mapping method. HPM datasets was obtained from our work and other datasets obtained from ProLinks database [13]. Numbers in small brackets denote interaction pair number in each dataset.

In protein interaction networks, the term degree represents the number of proteins that interact with a given target protein. The most highly connected proteins are usually the most important [15] and are considered to participate extensively in cellular processes. Among the twenty most highly connected proteins, most were found to be molecular chaperones, ribosomal proteins and ABC transporters (see Additional file 2). Molecular chaperones or ribosomal proteins are especially critical for the organism because of their extensive functional importance; for example, molecular chaperones help proteins to fold exactly and degrade those that fold incorrectly. This is extremely important for intracellular pathogens especially when they encounter environmental stresses imposed by host tissues. It is essential for *M. tuberculosis *to be able to resist damage from various pressures exerted by the host cells, which makes molecular chaperones especially critical for the survival of *M. tuberculosis *in its host.

In this respect, it is notable that molecular chaperones of *M. tuberculosis *have been reported to differ from those of other species [16]. For example, *M. tuberculosis *has two groEL genes, located in distinct regions of its genome, while most eubacteria contain only a single copy of this gene. The protein with the highest degree of connection to both groEL genes was Rv0685 (Tuf) and this protein showed increased expression inside macrophages in the BCG strain [17]. This also implicates molecular chaperones as essential for the survival of *M. tuberculosis *in its host, suggesting that these may be potential new drug targets.

In contrast to what is known regarding molecular chaperones, the functional importance of ABC transporters has not well been addressed, although these proteins account for about 2.5% of the genome of *M. tuberculosis *[18,19]. Their high connections to other network proteins imply that these transporter proteins might have important, but as yet unknown, functions in virulence signal transduction.

### Rv2752c is a metallo-beta-lactamase

**Figure 2:**
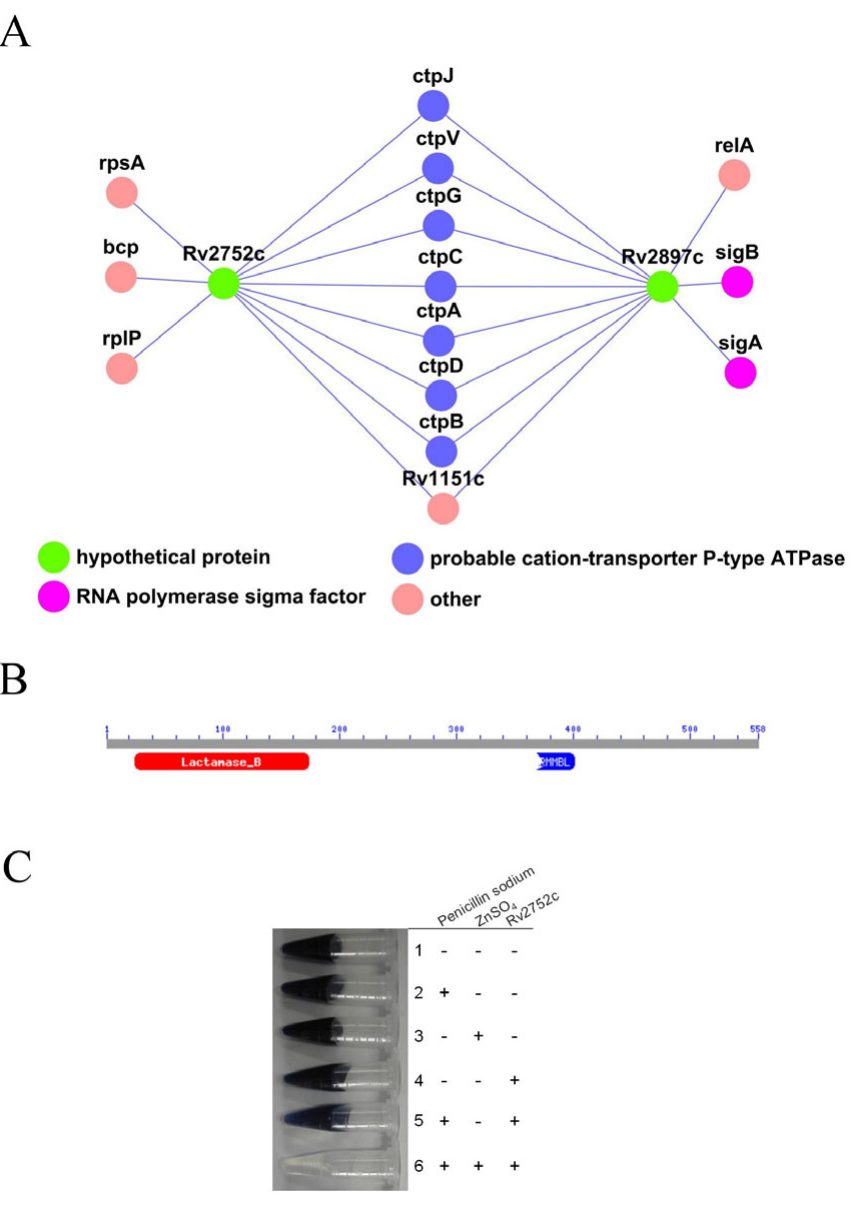
**A local PPI network of Rv2752c and its functional assay**. **(A) **A local PPI network of Rv2752c. **(B) **The domain structures of Rv2752c. (**C**) The activity assay of metallo-beta-lactamase for Rv2752c. The starch indicator solution and the iodine reagent were prepared. A solution containing 10,000 U of penicillin G per ml of phosphate buffer was freshly prepared and dispensed into small tubes. When a starch indicator was added to the mix with an iodine reagent, a blue color immediately developed due to the reaction of the iodine with the starch. The 0.6 mL reaction mixture contained 100 mM Tris-Cl (pH 7.5), 0.2 M NaCl, 100 ug/mL beta-lactamase, 1 mg/mL penicillin, 1% (m/v) starch, and I_2_/KI and with or without 10 mM ZnSO_4_. The reaction mixture was further rotated for up to six minutes at room temperature. Rapid decolorization occurred if the penicillin was hydrolyzed by beta-lactamase, which indicated positive beta-lactamase activity.

One important application of the PPI is aid in predicting the function of proteins forming the network. Thirty-three proteins have been annotated as hypothetical proteins in the *M. tuberculosis *genome from NCBI; these also appeared in the PPI networks (Fig. 1B) (also see Additional file 3). Their contexts within the network provide a basis by which their potential function and possible roles in cellular processes can be dissected. For instance, Rv2752c appeared to be linked to several metal cation-transporting D-type ATPases, which suggested that it might also be a metal-related protein (Fig. 2A). Using the CDD database [20], a metallo-beta-lactamase fold was found in Rv2752 that contained five sequence motifs. A lactamase B domain (pfam00753), which is common to all metallo-beta-lactamase, was found in the first four motifs of Rv2752 (Fig. 2B). The fifth motif, RMMBL (pfam07521), existed as a partial metallo-beta-lactamase and appeared to be specific to the function of the protein. Therefore, the existence of this RMMBL domain in Rv2752c implied that it was a typical metallo-beta-lactamase.

Previous research has demonstrated that the expression of metal-β-lactamases is one of the mechanisms by which bacterial drug resistances is imparted [21]. For *M. tuberculosis*, the activity of its metallo-beta-lactamase was examined using the purified protein following expression of Rv2752c in *E. coli*. The activity of the metallo-beta-lactamase was detected using a color reaction experiment and no activity was observed when the enzyme or metal ion were removed from the reactions (Fig. 2C).

Rv2752c and Rv2897c appeared to share the same context in local networks (Fig. 2A). Both were linked to several metal cation-transporting D-type ATPases. A Mg-chelatase domain (pfam01078) was also found in Rv2879c, which leads us to propose that the Rv2879c gene codes for another metallo-beta-lactamase.

### *M. tuberculosis *Ser/Thr protein kinases

**Figure 3:**
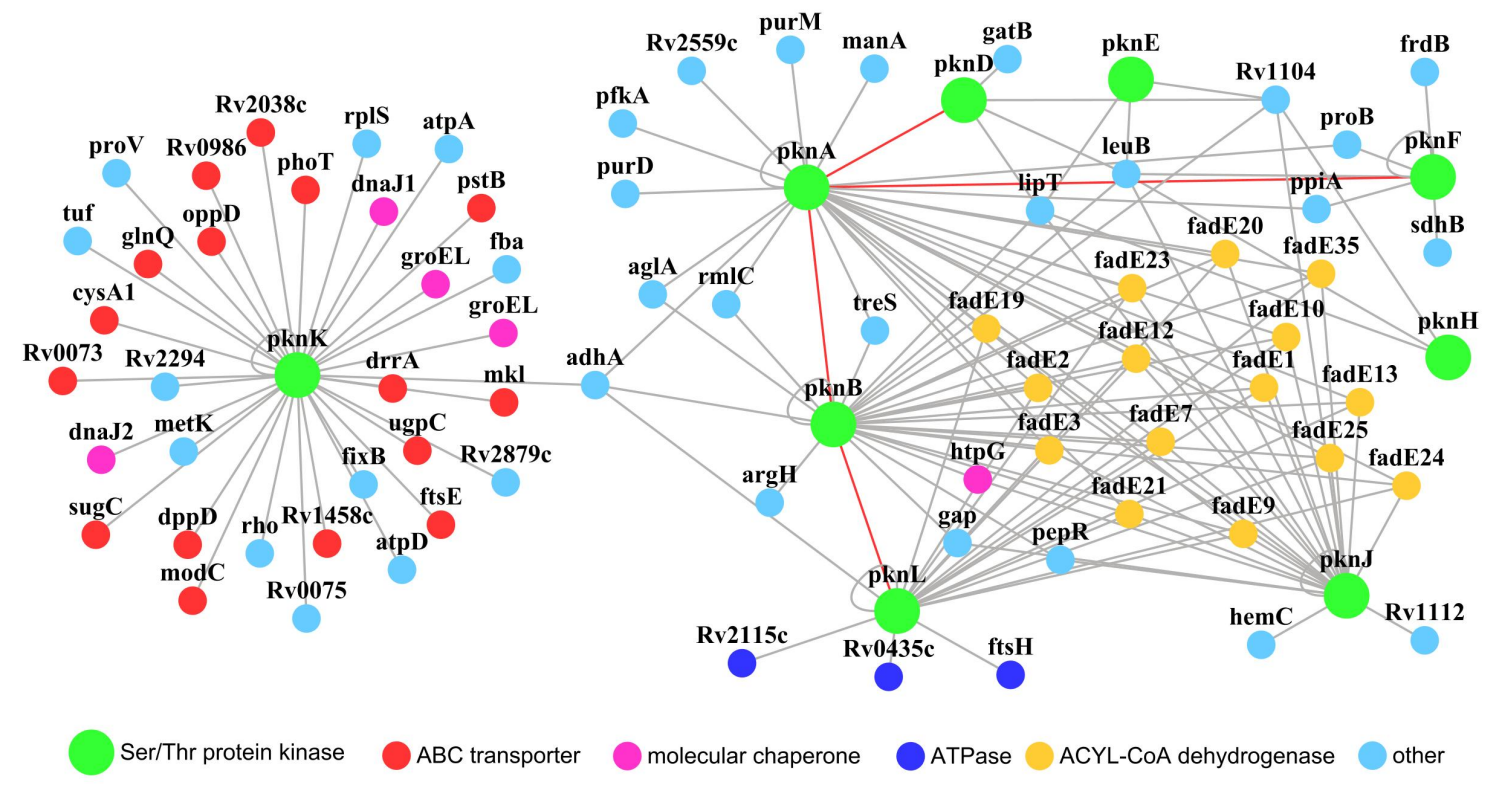
**A PPI network of eukaryotic-like Ser/Thr protein kinases**. The Protein interaction network was visualized using Cytoscape [42]. Nodes represent individual proteins and are linked with edges if they interacted with one another. Classes of proteins are colored differently as indicated.

The completion of the *M. tuberculosis *genome-sequencing project has now provided a complete list of 11 eukaryotic-like Ser/Thr protein kinases that form the *M. tuberculosis *STPK family [22]. These protein kinases appear to have important roles during infection [23,24]. Nine of these were found in the PPI networks of the current study (Fig. 3).

Ser/Thr protein kinases of *M. tuberculosis *participate in cell signaling processes such as cell division [23], fatty acid metabolism [24] and response to nitric oxide stress [25]. In the current study, two protein kinases, PknK and PknL, interacted with two cell division proteins, FtsE and FtsH, respectively, in the PPI network (Fig. 3). In addition, four network kinases, PknA, PknB, PknJ and PknL, were found to interact with a group of FadE proteins, indicating a potential regulatory role in fatty acid metabolism. Additionally, interactions were found between kinases such as PknA and PknB, and PknB and PknL, suggesting that co-regulation may also be occurring in the Ser/Thr protein kinases signal system.

PknK, although its function has not yet been established, appeared to extensively interact with the inner membrane-associated ATPase subunits of the network ABC transporters (Fig. 3). Unlike most protein kinases, PknK does not have transmembrane domains although it features a long regulatory structure in its C-terminus [26]. A previous study on protein localization showed that PknK might be associated with the inner membrane [27]. If so, the PknK may be able to participate in signal transductions in the nearby inner membrane by interacting with ABC transporters during infection.

### Rv1354c is a potential signal protein associated with ABC transporters

**Figure 4:**
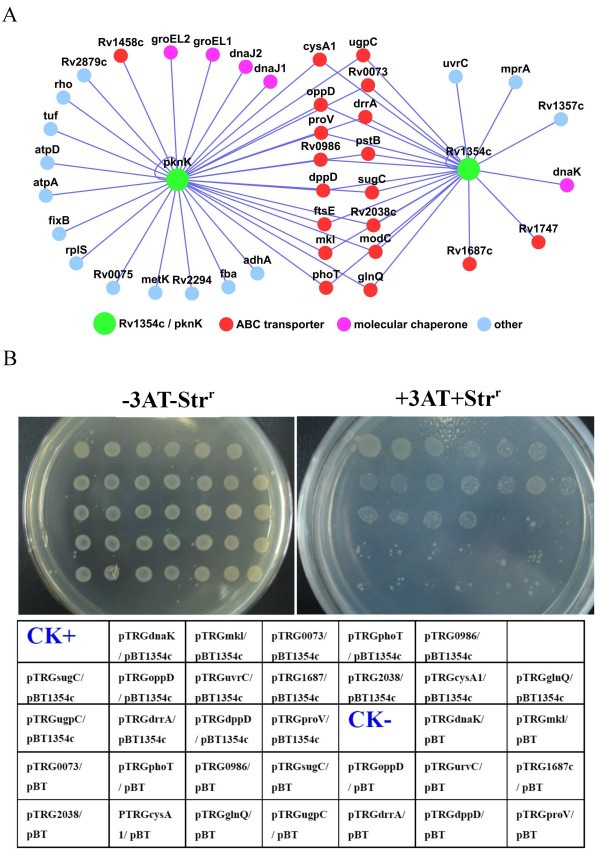
**(A) A PPI network of both PknK and Rv1354c with the same group of ABC transporters**. Nodes represent individual proteins and are linked with edges if they interacted with one another. Classes of proteins are colored differently as indicated. (B) Bacterial analysis of protein-protein interactions. Left panel, plate minus streptomycin (str) and 5 mM 3-amino-1,2,4-triazole (3-AT). Right panel, plate plus str and 5 mM 3-AT. An outline of the plates represents pairs of cotransformant strains. CK^+^, a cotransformant containing pBT-LGF2 and pTRG-Gal11^P ^as a positive control. CK^-^, cotransformant containing pBT and pTRG as a negative control.

Rv1354c is one of thirty-three hypothetical proteins included in the PPI network (Fig. 1B). Network searching revealed that Rv1354c interacted with essentially the same group of ABC transporter ATPase subunits as did PknK (Fig. 4A). Subcellular localization studies have revealed that Rv1354c is also associated with the inner membrane of *M. tuberculosis *[27]. This is consistent with the fact that no transmembrane region can be found in the protein when analyzed by using TMHMM  or SOSUI [28]. Thus, it implies that Rv1354c may localize near to the inner membrane through interactions with inner membrane proteins, such as the ATPase subunits of the ABC transporters. To confirm the interactions between Rv1354c and these proteins, a bacterial two-hybrid assay was conducted. Most of the contransformants grew well on the screening medium and no self-activation was observed (Fig. 4B). Therefore, Rv1354c was apparently interacting with the ABC transporters included in the PPI network (Fig. 4A).

**Figure 5:**
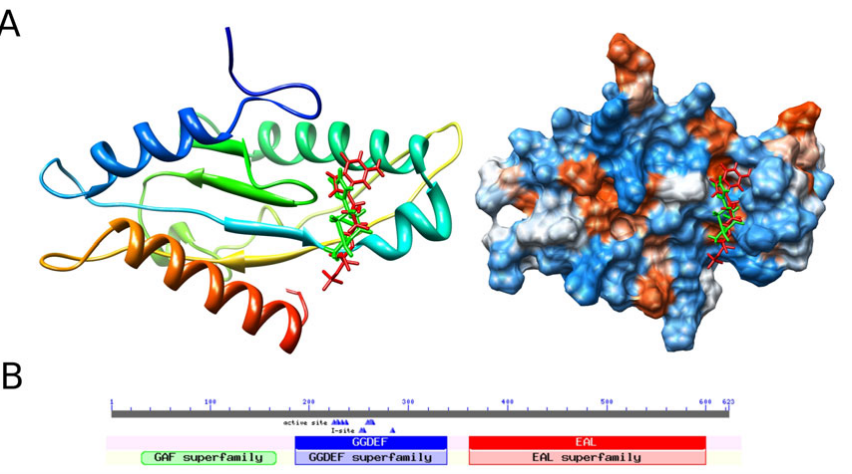
**(A) Structure-based inhibitor design of Rv1354c**. The structure of the GGDEF domain was obtained using the homology modeling method. The binding conformation of the GGDEF domain with GTPαS (red) and ZINC04632254 (green) from the docking is indicated in a different color (More compounds are listed in Additional file 4). **(B) **Schematic diagram of the Rv1354 domain structure.

Further domain analysis revealed that Rv1354c contained two typical domains of GGDEF and EAL (Fig. 5B). Both of these are involved in the turnover of cyclic-di-GMP, a multi-functional second messenger molecule in bacteria. The GGDEF domain exhibited diguanylate cyclase (DGC) activity, which is involved in the synthesis of cyclic-di-GMP from two GTPs. The EAL domain had the phosphodiesterase (PDE) activity required for the hydrolysis of cyclic-di-GMP [29]. Additionally, a sense domain GAF (E-value 1e-04) was also found in the N-terminus of Rv1354c. It has been proposed that the GAF structure has a role in sensing NO [30], a signal molecule involved in mediating the antimicrobial activity of macrophages [31].

An Rv1354 homologous protein from the *M. smegmatis *has been shown recently to have both *in vitro *diguanylate cyclase (DGC) and phosphodiesterase (PDE) activity [32]. Therefore, Rv1354c might be a unique signal protein and possibly is an essential component of the cyclic-di-GMP signaling system in *M. tuberculosis*.

### Structure-based inhibitor design for Rv1354c

The cyclic-di-GMP signal system is ubiquitous to bacteria but is absent in archaea and eukaryotes, making this an ideal system to target for potential therapeutic drugs. It also provides a basis for a selective inhibitor design. The Rv1354c appears to be a unique gene in *M. tuberculosis *that contains GAF-GGDEF-EAL domains and interacts with membrane-associated ABC transporters. Thus, Rv1354c may play an important role in sensing extracellular signals and in regulating the transcription of the *M. tuberculosis *genes. Additionally, Rv1354c is localized near to the inner membrane [27], a location that renders it relatively accessible to drugs. Taken together, the data indicate that Rv1354c may be an ideal target for the design of an anti-tuberculosis drug.

The biochemical and genetic functions of Rv1354c in *M. tuberculosis *have not yet been identified experimentally. For this reason, a structure-based inhibitor screening was performed, which targeted the GGDEF domain to inhibit its cyclic-di-GMP synthesis activity. At present, the 3D structure of Rv1354c is not known. Using the automated comparative protein modeling web server SWISS-MODEL [33] and the PleD protein (PDB ID: 2V0N) from *Caulobacter vibrioides *as a template (Identities = 41%), a structural modeling of the GGEDF domain of Rv1354c (Fig. 5) was performed. The 2VON is the structure of the PleD in complex with cyclic-di-GMP and GTPαS, which provides critical information for defining the active site. In addition, a docking-based virtual screening using DOCK6.1 [34] was designed. The Table S4 lists the top 10 hits (see Additional file 4). This provides the basis for development of new anti-tuberculosis drugs.

## Discussion

*M. tuberculosis *has been a major killer throughout history. Currently, it is still responsible for the deaths of about two million people each year. This unusual pathogenicity suggests that this mycobacterium may use unique mechanisms to facilitate an integrated response to the multiple stresses encountered upon entry into the phagosome, as well as for triggering some as-yet-to-be-identified switches to regulate the different phases of infection. Little is known regarding the molecular mechanisms of how extracellular signals may be sensed by *M. tuberculosis *during the infection process. The analysis of the pathogen interactome is a powerful approach for dissecting potential virulence pathways in order to explore new drug targets. In this study, we have described an approach, HPM, which allows the interactome of *M. tuberculosis *to be obtained based on the experimentally verified interactions from DIP (see Additional file 5). We constructed a comprehensive protein-protein interaction network for *M. tuberculosis*, which, upon further analysis, unraveled the potential function of hypothetical proteins and a probable signaling pathway. However, these methods, because they rely on sequence homology, can still give false positive results. To reduce this risk, we have comprehensively evaluated each query protein and its interaction using the e-value criteria in combination with the coverage and the sequence identity of the query to the hit protein (see Additional file 6).

In recent years, cyclic-di-GMP has been reported as a second messenger [29,35,36]. This signal system is involved in the regulation of a number of complex physiological processes in numerous pathogenic bacteria, including biofilm formation and virulence factor production [29,35,36]. However, the role of this signal system in *M. tuberculosis *is yet to be characterized. In this study, we found that Rv1354c is a new and unique signal protein that contains GAF-GGDEF-EAL domains and interacts with membrane-associated ABC transporters. Given its characteristics, Rv1354c may play an important role in sensing extracellular signals and in regulating the transcription of *M. tuberculosis *genes. This study's analysis strongly suggests that Rv1354c should be considered an essential component of the cyclic-di-GMP signaling system in *M. tuberculosis*. Based on the evidence from the current study, membrane-associated ABC transporters, a protein kinase signal, and a cyclic-di-GMP signaling system may be coordinating to form an integrated signaling pathway that allows *M. tuberculosis *to respond to extracellular stresses.

With the emergence of multi-drug resistant TB and co-infection with HIV, the incidence of tuberculosis has been increasing substantially worldwide over the past decade. However, no tuberculosis-specific drug has been discovered for more than 40 years. Analysis of the pathogen protein-protein interaction is a powerful approach for dissecting potential signal transduction and virulence pathways [37,38]. It also offers opportunities for exploring new drug targets. Two previous studies proposed the existence of a protein interaction network in *M. tuberculosis *based on the subcellular protein profiling [27] and gene expression profiling [39]. A very recent study discussed the potential pathways to drug resistance based on PPI analysis [40]. The current study suggests that the signal proteins of ABC transporters, PknK and Rv1354c may have previously unknown functions and probably essential roles for the *in vivo *growth of *M. tuberculosis*. As potential targets for drug development, these new proteins are attractive because they are localized near the inner membrane. In particular, the cyclic-di-GMP signaling transduction protein is apparently absent in human and mammalian cells and is essential for the virulence and stress resistance of *M. tuberculosis*. In this study, we found for the first time that Rv1354c contains multiple important signaling structures including GAF, GGDEF, and EAL domains, and that it interacts with membrane-associated ABC transporters. Thus, as a unique cyclic-di-GMP signaling transduction gene in *M. tuberculosis*, Rv1354c may play an important role in sensing extracellular signals that facilitate integrated responses to multiple stresses encountered by *M. tuberculosis *within the phagosome. Rv1354c therefore represents an ideal target for design of an anti-tuberculosis drug and further research should now focus on active site and docking-based inhibitors as potential new chemical weapons against the persistent pathogen *M. tuberculosis*.

## Conclusion

In this study, we comprehensively analyzed a PPI network that contained 738 proteins and 5639 non-redundant interaction pairs. A further analysis of this network unraveled the function of hypothetical proteins as well as a potential signaling pathway. A hypothetical protein Rv2752c was characterized as a metal-beta-lactamase through a domain analysis and *in vitro *activity experiment. Rv1354 was proposed to be responsible for the turnover of cyclic-di-GMP in the bacterium. Its interactions with a group of inner membrane-associated ABC transporters were confirmed by a bacterial two-hybrid analysis. Our analysis suggests that the coordination of a group of ATPase subunits of the ABC transporters, PknK and Rv1354c constitute a potential membrane-associated signaling pathway and all represent potential drug targets. The PPI network developed in this study, coupled with the findings of potential signal proteins, provides a basis for understanding the potential virulence and infection mechanisms of *M. tuberculosis*. They also suggest areas of focus in the search for new drug targets.

## Methods

### Bacterial strains, plasmids, enzymes, primers, and chemicals

*Escherichia coli *BL21 cells, purchased from Novagen, were used as the host strains to express *M. tuberculosis *proteins. The pBT, pTRG vectors and *Escherichia coli *host strains were purchased from Stratagene. The pET28a was purchased from Novagen. Restriction enzymes, T4 ligase, modification enzymes, Pyrobest DNA polymerase, deoxynucleoside triphosphates (dNTPs) and all antibiotics were obtained from TaKaRa Biotech. All reagents for the two-hybrid assay were purchased from Stratagene, while all PCR primers were synthesized by Invitrogen (Table S1). DNA purification kits were purchased from Watson Biotechnologies, and the Ni-NTA (Ni^2+^-nitrilotriacetate) agarose columns were obtained from Qiagen.

### Homologous protein mapping

To integrate accumulative protein interaction information obtained from the high throughput experiment, a computational method called homologous protein mapping (HPM) was used to predict *M. tuberculosis *protein interactions at the proteomic scale. With a pair of interactive proteins validated experimentally in the Database of Interaction Protein (DIP) [10], two proteins were predicted to have a functional linkage if they demonstrated higher homology. The schematic diagram of the HPM method is a simplified model in which a protein corresponds to just a single protein (Fig. 1A). However, when the homologous protein is actually defined, one protein may have a high homology in relation to more than one protein. In this case, a permutation strategy to create all possible interaction pairs is then used. For example, given that A and B have been validated experimentally, if protein A is homologous to C and D, then protein B is homologous to E and F. Consequently, the final mapping result will be composed of interaction pairs of CE, CF, DE and DF (Fig. 1A).

### Construction of a PPI network with HPM

The information collected in DIP as source data were selected because they integrated many types of evidence on protein-protein interactions including those from several model organisms [10].

The homology of proteins was assigned using the sequence similarity searching tool, ***blast ***[41]. The latest ***blast ***package (2.2.16) and amino acid sequence of *M. tuberculosis *were downloaded from the FTP Web site of NCBI. Using these amino acid sequence as input, a local blast database was subsequently constructed using the program ***formatdb ***released in the ***blast ***package. The amino acid sequence of proteins in the DIP database was downloaded from the DIP Web site (update to 20071007). Subsequently, all of the DIP amino acid sequence was used in the query. Then, the previous blast database was employed as target database to perform a large-scale blast search using ***blastp ***(E-value 1e-20). The preliminary blast results were further processed with our custom script for a convenient mapping procedure in the next step. Consequently, the linkage between these two sets of proteins was procured. Thereafter, the mapping process was completed according to the strategy described above. The Protein interaction network was visualized using Cytoscape [42].

### Expression, purification and β-lactamase activity analysis of the Rv2752 protein

The vectors expressing mycobacterial protein were constructed using the pET28a vector and primers described in Additional file 7. *Escherichia coli *BL21 (Novagen) was used as the host strain to express and purify proteins as described in a previously published procedures [43]. Purified proteins were greater than 99% pure as determined by SDS-PAGE. The beta-lactamase assay was also performed as previously described [44]. The starch indicator solution and the iodine reagent were prepared. A solution containing 10,000 U of penicillin G per ml of phosphate buffer was freshly prepared and dispensed in small tubes. When the starch indicator was added to the mix with the iodine reagent, a blue color immediately developed due to the reaction of the iodine with the starch. The 0.6 mL reaction mixture contained 100 mM Tris-Cl (pH 7.5), 0.2 M NaCl, 100 μg/mL beta-lactamase, 1 mg/mL penicillin, 1% (m/v) starch, and I_2_/KI and with or without 10 mM ZnSO_4_. The reaction mixture was further rotated for up to six minutes at room temperature. Rapid decolorization occurred if the penicillin was hydrolyzed by beta-lactamase, which indicated positive beta-lactamase activity.

### Bacterial two-hybrid analysis of protein-protein interactions

The BacterioMatch II Two-Hybrid System Library Construction Kit (Stratagene) was used to detect protein-protein interactions between mycobacterial proteins. Bacterial two-hybrid analysis was carried out according to the manufacturer's directions. The pBT and pTRG vectors containing mycobacterial genes were generated. All primers used for PCR amplification are described in Additional file 7. Positive growth cotransformants were selected on the Selective Screening Medium plate containing 5 mM 3-AT (Stratagene), 8 μg/ml streptomycin, 15 μg/ml tetracycline, 34 μg/ml chloramphenicol, and 50 μg/ml kanamycin. A cotransformant containing pBT-LGF2 and pTRG-Gal11P (Stratagene) was used as a positive control for expected growth on the Selective Screening Medium. A cotransformant containing empty vector pBT and pTRG was also used as a negative control.

### Homology modeling and docking based virtual screening

According to protein interaction information and domain structures, we propose that Rv1354c is a suitable target for new anti-tuberculosis drugs, and a docking-based inhibitor screening was performed *in silicon*. The structure of the GGDEF domain of Rv1354c was modeled computationally using the automated comparative protein modeling web server SWISS-MODEL [33]. Likewise, the docking-based virtual screening was performed using DOCK [34]. The compound library was obtained from the ZINC that supplied database molecules as 3D formats that were ready-to-dock [45].

## Abbreviations

PPI: Protein-Protein Interaction; HPM: Homologous Protein Mapping; DIP: Database of Interaction Protein; TB: Tuberculosis; DGC: Diguanylate Cyclase; PDE: Phosphodiesterase.

## Competing interests

The authors declare that they have no competing interests.

## Authors' contributions

TC performed data analysis and wrote the manuscript. LZ and XW performed the experiments. ZGH performed the data analysis, helped in the experimental design, supervised the experiments, and wrote the manuscript. All authors read and approved the final manuscript.

## Supplementary Material

Additional file 1 **A protein-protein interaction network of *M. tuberculosis *H37Rv. **The data provided represent the protein interaction network of *M. tuberculosis *H37Rv generated from HPM method which composed with 793 individual proteins and 6091 interaction pairs. Click here for file

Additional file 2 **Top 20 highly connected Nodes in the network. **The data provided represent the top 20 highly connected Nodes in the network. Click here for file

Additional file 3 **Hypothetical proteins in the network. **The data present the hypothetical proteins in the network. Click here for file

Additional file 4 **Top 10 hits compounds in structure based virtual screen. **The data provided top 10 hits compounds in structure based virtual screen. Click here for file

Additional file 5 **The interactome of *Mycobacterium tuberculosis *obtained based on the experimentally verified interactions from DIP.** The data present all protein-protein interactions of *Mycobacterium tuberculosis *obtained based on the experimentally verified interactions from DIP. Click here for file

Additional file 6 **Identity, coverage and E-value of each query protein of *Mycobacterium tuberculosis *to the hit protein in DIP. **The data described the identity, coverage and E-value of each query protein of *Mycobacterium tuberculosis *to the hit protein in DIP. Click here for file

Additional file 7 **Primers and plasmids used in the study. **The data present the primers and plasmids used in the study. Click here for file
