# Supplementary material for: Uncovering new signaling proteins and potential drug targets through the interactome analysis of Mycobacterium tuberculosis
Source: BMC Genomics. 2009 Mar 19;10:118. doi: 10.1186/1471-2164-10-118 (PMC2671525; doi:10.1186/1471-2164-10-118)
Supplement: Additional file 1 — A protein-protein interaction network of M. tuberculosis H37Rv. The data provided represent the protein interaction network of M. tuberculosis H37Rv generated from HPM method which composed with 793 individual proteins and 6091 interaction pairs. [file 1471-2164-10-118-S1.doc]

**Additional file 1**

**Fig S1**

**
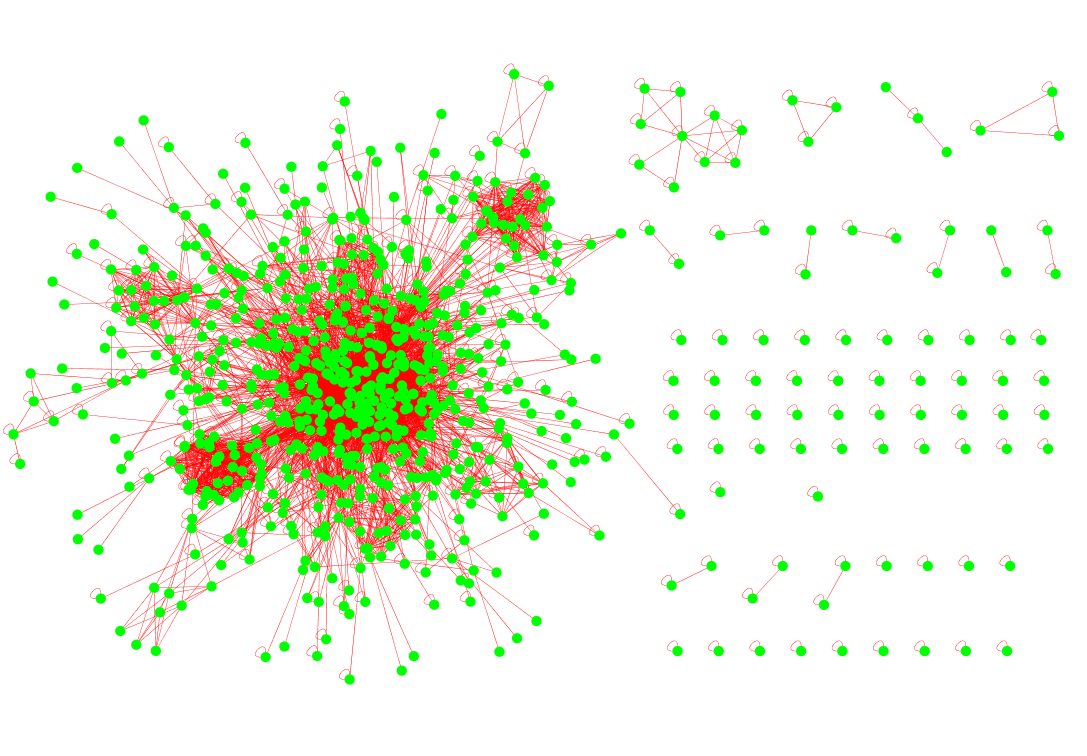
**

**A protein-protein interaction network of *M. tuberculosis* H37Rv.** Protein interaction network of *M. tuberculosis* H37Rv generated from HPM method which composed with 793 individual proteins and 6091 interaction pairs.
