# Supplementary material for: Uncovering new signaling proteins and potential drug targets through the interactome analysis of Mycobacterium tuberculosis
Source: BMC Genomics. 2009 Mar 19;10:118. doi: 10.1186/1471-2164-10-118 (PMC2671525; doi:10.1186/1471-2164-10-118)
Supplement: Additional file 2 — Top 20 highly connected Nodes in the network. The data provided represent the top 20 highly connected Nodes in the network. [file 1471-2164-10-118-S2.doc]

**Additional file 2**

**Table S1. Top 20 highly connected Nodes in the network**.

|  | **Synom** | **gene** | **degree** | **Function** |
| --- | --- | --- | --- | --- |
| 1 | Rv0685 | tuf | 130 | elongation factor |
| 2 | Rv0350 | **dnaK** | 124 | molecular chaperone |
| 3 | Rv2890c | **rpsB** | 111 | 30S ribosomal protein S2 |
| 4 | Rv3417c | **groEL1** | 99 | chaperonin |
| 5 | Rv0721 | **rpsE** | 99 | 30S ribosomal protein S5 |
| 6 | Rv0440 | **groEL2** | 99 | chaperonin |
| 7 | Rv1253 | deaD | 97 | ATP-dependent RNA helicase |
| 8 | Rv3211 | rhlE | 96 | probable ATP-dependent RNA helicase |
| 9 | Rv2397 | **cysA1** | 94 | probable sulfate-transport ABC transporter |
| 10 | Rv0702 | **rplD** | 87 | 50S ribosomal protein L4 |
| 11 | Rv0667 | rpoB | 83 | DNA-directed RNA polymerase beta subunit |
| 12 | Rv0655 | **mkl** | 81 | probable ribonucleotide-transport ABC transporter |
| 13 | Rv0706 | **rplV** | 80 | 50S ribosomal protein L22 |
| 14 | Rv0683 | **rpsG** | 73 | 30S ribosomal protein S7 |
| 15 | Rv3596c | clpC1 | 72 | probable ATP-dependent protease ATP-binding subunit |
| 16 | Rv0384c | **clpB** | 72 | heat shock protein |
| 17 | Rv3663c | **dppD** | 71 | probable dipeptide-transport ABC transporter |
| 18 | Rv2855 | mtr | 70 | dihydrolipoamide dehydrogenase |
| 19 | Rv2713 | sthA | 70 | soluble pyridine nucleotide transhydrogenase |
| 20 | Rv0933 | **pstB** | 70 | phosphate-transport ABC transporter |
