# Supplementary material for: Uncovering new signaling proteins and potential drug targets through the interactome analysis of Mycobacterium tuberculosis
Source: BMC Genomics. 2009 Mar 19;10:118. doi: 10.1186/1471-2164-10-118 (PMC2671525; doi:10.1186/1471-2164-10-118)
Supplement: Additional file 3 — Hypothetical proteins in the network. The data present the hypothetical proteins in the network. [file 1471-2164-10-118-S3.doc]

**Additional file 3**

**Table S3. Hypothetical proteins in the network**

| Hypothetical protein | Interactive partner | Hypothetical protein | Interactive partner | Hypothetical protein | Interactive partner | Hypothetical protein | Interactive partner |
| --- | --- | --- | --- | --- | --- | --- | --- |
| Rv0118c | Rv0118c | Rv0118c | Rv3417c | Rv1354c | Rv2397c | Rv1422 | Rv2448c |
| Rv0118c | Rv0440 | Rv0118c | Rv3443c | Rv1354c | Rv2564 | Rv1422 | Rv2688c |
| Rv0118c | Rv0462 | Rv0118c | Rv3456c | Rv1354c | Rv2832c | Rv1422 | Rv2713 |
| Rv0118c | Rv0641 | Rv0118c | Rv3458c | Rv1354c | Rv2936 | Rv1422 | Rv2748c |
| Rv0118c | Rv0667 | Rv0118c | Rv3460c | Rv1354c | Rv3102c | Rv1422 | Rv2855 |
| Rv0118c | Rv0668 | Rv0118c | Rv3470c | Rv1354c | Rv3663c | Rv1422 | Rv2890c |
| Rv0118c | Rv0672 | Rv0118c | Rv3797 | Rv1354c | Rv3758c | Rv1422 | Rv2936 |
| Rv0118c | Rv0683 | Rv0566c | Rv0002 | Rv1357c | Rv0350 | Rv1422 | Rv3102c |
| Rv0118c | Rv0701 | Rv0566c | Rv0566c | Rv1357c | Rv1354c | Rv1422 | Rv3240c |
| Rv0118c | Rv0702 | Rv0647c | Rv0647c | Rv1357c | Rv1357c | Rv1422 | Rv3272 |
| Rv0118c | Rv0704 | Rv0647c | Rv3676 | Rv1421 | Rv0440 | Rv1422 | Rv3303c |
| Rv0118c | Rv0706 | Rv0727c | Rv0727c | Rv1421 | Rv0685 | Rv1422 | Rv3443c |
| Rv0118c | Rv0707 | Rv0858c | Rv0733 | Rv1421 | Rv0706 | Rv1422 | Rv3758c |
| Rv0118c | Rv0708 | Rv0858c | Rv1394c | Rv1421 | Rv1421 | Rv1461 | Rv1461 |
| Rv0118c | Rv0714 | Rv0858c | Rv1547 | Rv1421 | Rv3417c | Rv1461 | Rv1463 |
| Rv0118c | Rv0716 | Rv0858c | Rv3370c | Rv1422 | Rv0462 | Rv1711 | Rv0118c |
| Rv0118c | Rv0719 | Rv1003 | Rv1003 | Rv1422 | Rv0655 | Rv1711 | Rv0641 |
| Rv0118c | Rv0721 | Rv1003 | Rv2215 | Rv1422 | Rv0667 | Rv1711 | Rv0655 |
| Rv0118c | Rv0794c | Rv1003 | Rv2495c | Rv1422 | Rv0683 | Rv1711 | Rv0672 |
| Rv0118c | Rv1253 | Rv1021 | Rv0440 | Rv1422 | Rv0701 | Rv1711 | Rv0683 |
| Rv0118c | Rv1415 | Rv1021 | Rv1021 | Rv1422 | Rv0702 | Rv1711 | Rv0685 |
| Rv0118c | Rv1630 | Rv1021 | Rv2364c | Rv1422 | Rv0706 | Rv1711 | Rv0700 |
| Rv0118c | Rv1643 | Rv1021 | Rv3417c | Rv1422 | Rv0721 | Rv1711 | Rv0701 |
| Rv0118c | Rv1711 | Rv1063c | Rv1063c | Rv1422 | Rv0758 | Rv1711 | Rv0702 |
| Rv0118c | Rv1820 | Rv1354c | Rv0073 | Rv1422 | Rv0794c | Rv1711 | Rv0704 |
| Rv0118c | Rv1821 | Rv1354c | Rv0350 | Rv1422 | Rv0855 | Rv1711 | Rv0706 |
| Rv0118c | Rv1940 | Rv1354c | Rv0655 | Rv1422 | Rv1143 | Rv1711 | Rv0707 |
| Rv0118c | Rv2215 | Rv1354c | Rv0820 | Rv1422 | Rv1164 | Rv1711 | Rv0716 |
| Rv0118c | Rv2241 | Rv1354c | Rv0933 | Rv1422 | Rv1213 | Rv1711 | Rv0719 |
| Rv0118c | Rv2495c | Rv1354c | Rv0981 | Rv1422 | Rv1218c | Rv1711 | Rv0721 |
| Rv0118c | Rv2583c | Rv1354c | Rv0986 | Rv1422 | Rv1238 | Rv1711 | Rv1095 |
| Rv0118c | Rv2713 | Rv1354c | Rv1238 | Rv1422 | Rv1422 | Rv1711 | Rv1218c |
| Rv0118c | Rv2839c | Rv1354c | Rv1281c | Rv1422 | Rv1458c | Rv1711 | Rv1238 |
| Rv0118c | Rv2855 | Rv1354c | Rv1354c | Rv1422 | Rv1687c | Rv1711 | Rv1253 |
| Rv0118c | Rv2890c | Rv1354c | Rv1357c | Rv1422 | Rv1736c | Rv1711 | Rv1458c |
| Rv0118c | Rv2904c | Rv1354c | Rv1420 | Rv1422 | Rv1747 | Rv1711 | Rv1551 |
| Rv0118c | Rv3003c | Rv1354c | Rv1687c | Rv1422 | Rv1821 | Rv1711 | Rv1630 |
| Rv0118c | Rv3211 | Rv1354c | Rv1747 | Rv1422 | Rv1866 | Rv1711 | Rv1687c |
| Rv0118c | Rv3240c | Rv1354c | Rv1859 | Rv1422 | Rv2241 | Rv1711 | Rv1711 |
| Rv0118c | Rv3303c | Rv1354c | Rv2038c | Rv1422 | Rv2397c | Rv1711 | Rv1747 |
| **Hypothetical protein** | **Interactive partner** | **Hypothetical protein** | **Interactive partner** | **Hypothetical protein** | **Interactive partner** | **Hypothetical protein** | **Interactive partner** |
| Rv1711 | Rv1820 | Rv1820 | Rv0716 | Rv2522c | Rv3417c | Rv2879c | Rv0363c |
| Rv1711 | Rv2241 | Rv1820 | Rv0719 | Rv2603c | Rv0524 | Rv2879c | Rv0384c |
| Rv1711 | Rv2368c | Rv1820 | Rv0721 | Rv2603c | Rv0815c | Rv2879c | Rv0462 |
| Rv1711 | Rv2397c | Rv1820 | Rv0794c | Rv2603c | Rv1568 | Rv2879c | Rv0490 |
| Rv1711 | Rv2444c | Rv1820 | Rv1253 | Rv2603c | Rv1655 | Rv2879c | Rv0641 |
| Rv1711 | Rv2482c | Rv1820 | Rv1415 | Rv2603c | Rv2291 | Rv2879c | Rv0668 |
| Rv1711 | Rv2555c | Rv1820 | Rv1630 | Rv2603c | Rv2589 | Rv2879c | Rv0683 |
| Rv1711 | Rv2583c | Rv1820 | Rv1643 | Rv2603c | Rv2603c | Rv2879c | Rv0684 |
| Rv1711 | Rv2688c | Rv1820 | Rv1711 | Rv2603c | Rv3051c | Rv2879c | Rv0685 |
| Rv1711 | Rv2783c | Rv1820 | Rv1820 | Rv2603c | Rv3117 | Rv2879c | Rv0700 |
| Rv1711 | Rv2879c | Rv1820 | Rv1821 | Rv2603c | Rv3290c | Rv2879c | Rv0701 |
| Rv1711 | Rv2890c | Rv1820 | Rv1940 | Rv2603c | Rv3329 | Rv2879c | Rv0702 |
| Rv1711 | Rv2904c | Rv1820 | Rv2215 | Rv2604c | Rv2606c | Rv2879c | Rv0704 |
| Rv1711 | Rv2936 | Rv1820 | Rv2241 | Rv2674 | Rv0137c | Rv2879c | Rv0706 |
| Rv1711 | Rv3003c | Rv1820 | Rv2495c | Rv2674 | Rv0667 | Rv2879c | Rv0707 |
| Rv1711 | Rv3102c | Rv1820 | Rv2583c | Rv2674 | Rv0668 | Rv2879c | Rv0716 |
| Rv1711 | Rv3211 | Rv1820 | Rv2713 | Rv2674 | Rv1281c | Rv2879c | Rv0719 |
| Rv1711 | Rv3442c | Rv1820 | Rv2839c | Rv2674 | Rv1384 | Rv2879c | Rv0721 |
| Rv1711 | Rv3443c | Rv1820 | Rv2855 | Rv2674 | Rv2457c | Rv2879c | Rv0758 |
| Rv1711 | Rv3458c | Rv1820 | Rv2890c | Rv2674 | Rv2674 | Rv2879c | Rv0794c |
| Rv1711 | Rv3460c | Rv1820 | Rv2904c | Rv2674 | Rv3663c | Rv2879c | Rv1032c |
| Rv1711 | Rv3470c | Rv1820 | Rv3003c | Rv2718c | Rv0700 | Rv2879c | Rv1058 |
| Rv1711 | Rv3646c | Rv1820 | Rv3211 | Rv2718c | Rv2718c | Rv2879c | Rv1193 |
| Rv1711 | Rv3758c | Rv1820 | Rv3240c | Rv2752c | Rv0092 | Rv2879c | Rv1253 |
| Rv1711 | Rv3797 | Rv1820 | Rv3303c | Rv2752c | Rv0103c | Rv2879c | Rv1392 |
| Rv1820 | Rv0118c | Rv1820 | Rv3417c | Rv2752c | Rv0708 | Rv2879c | Rv1438 |
| Rv1820 | Rv0440 | Rv1820 | Rv3443c | Rv2752c | Rv0969 | Rv2879c | Rv1630 |
| Rv1820 | Rv0462 | Rv1820 | Rv3456c | Rv2752c | Rv1151c | Rv2879c | Rv1640c |
| Rv1820 | Rv0641 | Rv1820 | Rv3458c | Rv2752c | Rv1469 | Rv2879c | Rv1641 |
| Rv1820 | Rv0667 | Rv1820 | Rv3460c | Rv2752c | Rv1630 | Rv2879c | Rv1643 |
| Rv1820 | Rv0668 | Rv1820 | Rv3470c | Rv2752c | Rv1992c | Rv2879c | Rv1711 |
| Rv1820 | Rv0672 | Rv1820 | Rv3797 | Rv2752c | Rv2521 | Rv2879c | Rv1821 |
| Rv1820 | Rv0683 | Rv1841c | Rv1841c | Rv2752c | Rv3270 | Rv2879c | Rv2215 |
| Rv1820 | Rv0701 | Rv1842c | Rv3834c | Rv2752c | Rv3743c | Rv2879c | Rv2241 |
| Rv1820 | Rv0702 | Rv1866 | Rv1422 | Rv2786c | Rv0629c | Rv2879c | Rv2373c |
| Rv1820 | Rv0704 | Rv2148c | Rv0700 | Rv2786c | Rv2241 | Rv2879c | Rv2441c |
| Rv1820 | Rv0706 | Rv2148c | Rv2148c | Rv2786c | Rv2786c | Rv2879c | Rv2444c |
| Rv1820 | Rv0707 | Rv2205c | Rv1633 | Rv2879c | Rv0005 | Rv2879c | Rv2495c |
| Rv1820 | Rv0708 | Rv2205c | Rv2205c | Rv2879c | Rv0120c | Rv2879c | Rv2505c |
| Rv1820 | Rv0714 | Rv2522c | Rv0440 | Rv2879c | Rv0352 | Rv2879c | Rv2583c |

| **Hypothetical protein** | **Interactive partner** | **Hypothetical protein** | **Interactive partner** |
| --- | --- | --- | --- |
| Rv2879c | Rv2713 | Rv3329 | Rv2589 |
| Rv2879c | Rv2737c | Rv3329 | Rv2603c |
| Rv2879c | Rv2783c | Rv3329 | Rv3211 |
| Rv2879c | Rv2839c | Rv3329 | Rv3290c |
| Rv2879c | Rv2855 | Rv3329 | Rv3329 |
| Rv2879c | Rv2879c | Rv3329 | Rv3598c |
| Rv2879c | Rv2890c | Rv3433c | Rv0462 |
| Rv2879c | Rv2904c | Rv3433c | Rv0794c |
| Rv2879c | Rv2948c | Rv3433c | Rv1420 |
| Rv2879c | Rv3080c | Rv3433c | Rv2215 |
| Rv2879c | Rv3089 | Rv3433c | Rv2495c |
| Rv2879c | Rv3148 | Rv3433c | Rv2713 |
| Rv2879c | Rv3211 | Rv3433c | Rv2855 |
| Rv2879c | Rv3240c | Rv3433c | Rv3303c |
| Rv2879c | Rv3303c | Rv3433c | Rv3433c |
| Rv2879c | Rv3443c | Rv3433c | Rv3579c |
| Rv2879c | Rv3458c |  |  |
| Rv2879c | Rv3561 |  |  |
| Rv2879c | Rv3596c |  |  |
| Rv2879c | Rv3598c |  |  |
| Rv2879c | Rv3764c |  |  |
| Rv2897c | Rv0092 |  |  |
| Rv2897c | Rv0103c |  |  |
| Rv2897c | Rv0969 |  |  |
| Rv2897c | Rv1151c |  |  |
| Rv2897c | Rv1469 |  |  |
| Rv2897c | Rv1992c |  |  |
| Rv2897c | Rv2583c |  |  |
| Rv2897c | Rv2703 |  |  |
| Rv2897c | Rv2710 |  |  |
| Rv2897c | Rv3270 |  |  |
| Rv2897c | Rv3743c |  |  |
| Rv3272 | Rv1422 |  |  |
| Rv3292 | Rv3292 |  |  |
| Rv3329 | Rv1253 |  |  |
| Rv3329 | Rv1568 |  |  |
| Rv3329 | Rv1640c |  |  |
| Rv3329 | Rv1655 |  |  |
| Rv3329 | Rv2101 |  |  |
| Rv3329 | Rv2321c |  |  |
