# Supplementary material for: Uncovering new signaling proteins and potential drug targets through the interactome analysis of Mycobacterium tuberculosis
Source: BMC Genomics. 2009 Mar 19;10:118. doi: 10.1186/1471-2164-10-118 (PMC2671525; doi:10.1186/1471-2164-10-118)
Supplement: Additional file 4 — Top 10 hits compounds in structure based virtual screen. The data provided top 10 hits compounds in structure based virtual screen. [file 1471-2164-10-118-S4.doc]

**Additional file 4**

**Table S4**. Top 10 hits compounds in structure based virtual screen

| ZINC04632254 | 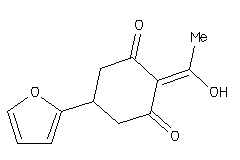 |
| --- | --- |
| ZINC04149400 | 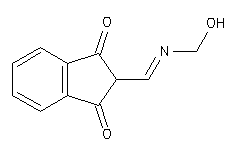 |
| ZINC00109814 | 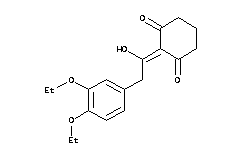 |
| ZINC05286123 | 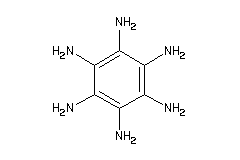 |
| ZINC04807335 | 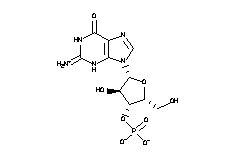 |
| ZINC02115188 | 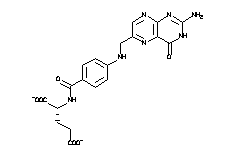 |
| ZINC04261903 | 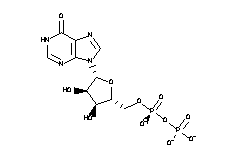 |
| ZINC04807333 | 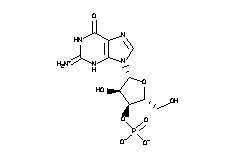 |
| ZINC04261904 | 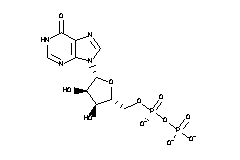 |
| ZINC00716686 | 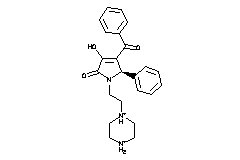 |
