# Supplementary material for: Uncovering new signaling proteins and potential drug targets through the interactome analysis of Mycobacterium tuberculosis
Source: BMC Genomics. 2009 Mar 19;10:118. doi: 10.1186/1471-2164-10-118 (PMC2671525; doi:10.1186/1471-2164-10-118)
Supplement: Additional file 7 — Primers and plasmids used in the study. The data present the primers and plasmids used in the study. [file 1471-2164-10-118-S7.doc]

**Additional file 7**

**Table S2.** Primers and plasmids used in the study

| Genes | Names | Primer sequences^a^ | Recombinant plasmids |
| --- | --- | --- | --- |
| Rv0073 |  | 5'-ATATGAATTCAGGTGGGCGATCTCAGCATTCA-3'  5'-ATATTCTAGAAGTTAGTCGTTGGCAAGCGCAC-3' | pTRG0073 |
| Rv0350 | *dnaK* | 5'-ATATGAATTCGAATGGCTCGTGCGGTCGGGAT-3'  5'-ATATTCTAGATCAGACCACCTCCGCGTCCACA-3' | pTRGdnaK |
| Rv0655 | *mkl* | 5'-ATATGAATTCGAGTGCGATACAGTGACTCATA-3'  5'-ATATTCTAGAATTTACTGGCCGATTTCGTGCA-3' | pTRGmkl |
| Rv0820 | *phoT* | 5'-ATCGGAATTCCTGTGGCCAAGCGGTTGGACCT-3'  5'-ATCGTATCTAGACTAGCCGAAGCGCCCGGAGA-3' | pTRGphoT |
| Rv0986 |  | 5'-ATATGAATTCAAATGAACCGGCAACCTATCGT-3'  5'-ATATTCTAGAGATCATTCATATGACGTGGGCA-3' | pTRG0986 |
| Rv1238 | *sugC* | 5'-CATCGAATTCGAATGGCCGAGATTGTGTTGGA-3'  5'-ATATTCTAGAATTTAGGCGCGGTGCGGAATGG-3' | pTRGsugC |
| Rv1281c | *oppD* | 5'-ATATGAATTCGAATGAGCCCCCTGCTCGAGGT-3'  5'-ATATATCTAGATCAGCCACGACGGGCCGGATC-3' | pTRGoppD |
| Rv1420 | *uvrC* | 5'-AGCGGAATTCATGTGCCAGATCCCGCAACGTA-3'  5'-ATATATTCTAGATCATCGCGCGGCCCCCGATG-3' | pTRGuvrC |
| Rv1687c |  | 5'-CGCGCGAATTCGCGTGATGATTTCATCAAGTG-3'  5'-TATATTCTAGAATTTAGCCGGCCGCGGGCACG-3' | pTRG1687 |
| Rv2038c |  | 5'-AGCTGAATTCGCATGGCTTCGGTGAGTTTTGA-3'  5'-ATATTCTAGAATTTAGCCCGGAATCCGACGCC-3' | pTRG2038 |
| Rv2397c | *cysA1* | 5'-ATATGAATTCAAATGACCTACGCCATCGTCGT-3'  5'-ATATTCTAGAGCTCACGTCGATGTGACTTTGA-3' | pTRGcysA1 |
| Rv2564 | *glnQ* | 5'-ATATGAATTCGAATGGGCGGCCTAACCATTTC-3'  5'-ATATTCTAGAATCTATTCGCTGGCAAGCTCGC-3' | pTRGglnQ |
| Rv2832c | *ugpC* | 5'-GACGGAATTCGCATGGCTAACGTTCAGTACTC-3'  5'-ATATATCTAGATCAGCGAAGCCGGGTCTCGGT-3' | pTRGugpC |
| Rv2936 | *drrA* | 5'-ATATGAATTCGCATGCGCAACGACGACATGGC-3'  5'-ATATATTCTAGATCATCGCGCGGACCCCGACA-3' | pTRGdrrA |
| Rv3663c | *dppD* | 5'-ATATGAATTCAAATGAGCGTCCCAGCAGCGCC-3'  5'-GATCTCTAGAGCTCACAGGTTGCCAACCTTTC-3' | pTRGdppD |
| Rv3758c | *proV* | 5'-ATGAGAATTCGATTGATCTGCTTTGACGATGT-3'  5'-ATATATTCTAGATCAGGTAGTGCATGGCTTAG-3' | pTRGproV |
| Rv1354c |  | 5'-ATATGCGGCCGCAATGTGCAACGACACCGCGA-3'  5'-GCGCCTCGAGTCAAGATAACGCCGGGTCAGTT-3' | pTRG1354 |
| Rv2752c |  | 5'-CCGCCCGAATTCGCGTGGATGTAGACCTTCCC-3'  5'-CCGCCCTCTAGATCACACCTCGATGACCGTCG-3' | pET2752 |

a, The sites for restriction enzymes are underlined.
